# Measuring professional identity in health and social care students: single-item question versus the Macleod Clark Professional Identity Scale (MCPIS-9)

**DOI:** 10.3389/fmed.2026.1926091

**Published:** 2026-09-24

**Authors:** Aslak Steinsbekk, Cathinka Thyness, Hilde Grimstad, Ragna Stalsberg

**Affiliations:** 1 Department of Public Health and Nursing, Faculty of Medicine and Health Sciences, Norwegian University of Science and Technology (NTNU), Trondheim, Norway; 2 Trondheim Municipality, Trondheim, Norway; 3 Department of Circulation and Medical Imaging, Faculty of Medicine and Health Sciences, Norwegian University of Science and Technology (NTNU), Trondheim, Norway

**Keywords:** higher education, measure, professional identity, single-item measures, student development

## Abstract

**Background:**

Existing measures of professional identity often yield high scores among students that may mask differences. One frequently used measure of professional identity is the one factor 9-item questionnaire MCPIS-9 with a five-point balanced answering scale. The aim of this study was to investigate the performance of the MCPIS-9 compared to a single-item question with five positively unbalanced response options.

**Methods:**

An explorative cross-sectional study inviting all students from all academic years in 11 health and social care programs at one university. Two instruments were used to assess professional identity (response options): the Norwegian version of the 9-item Macleod Clark Professional Identity Scale (MCPIS-9) (strongly disagree, disagree, neither agree nor disagree, agree, strongly agree), and a single-item question, “All things considered, to what extent do you identify with the profession you are training for?” (not at all, some¸ much, very much, completely). Data were analysed using descriptive statistics, Spearman correlation and multivariable regression.

**Results:**

A total of 864 students from 11 programs participated (mean age 24 years; 77% women). The MCPIS-9 had a mean score of 4.15 (standard deviation 0.54), and the single-item question lower mean score and twice the variation (mean 3.58, standard deviation 0.99). The two measures correlated (Spearman's *ρ* = 0.61, *p* < 0.001). Both detected higher professional identity among students in later years, with the single-item question having a large difference (0.68 point from first to final year, MCPIS-9 0.19 point). Multivariable models showed that both measures identified academic year and having the program as first choice, as being the variables most strongly associated with higher professional identity, with the single-item question having larger adjusted coefficients.

**Conclusion:**

The single-item measure showed comparable validity evidence to the MCPIS-9 and produced greater score dispersion in this sample. Given its simplicity and low respondent burden, it may be a useful alternative as and overall measure of professional identity.

## Introduction

Professional identity is a complex and multidimensional construct that has been conceptualised in broad and varied ways across the health professions (1, 2). It encompasses the integration of attitudes, values, knowledge, beliefs, and skills shared within a professional group (3–5). Multi-item scales are often considered necessary to capture the full range of professional identity development and ensure reliable and valid assessment (6). However, despite its recognised importance for healthcare quality and the delivery of effective and compassionate care (4), there is still no gold standard for measuring professional identity, and the evidence supporting existing instruments is limited and sometimes inconsistent (6, 7).

Several studies assessing professional identity among health and social care students using such scales, have reported high scores, often approaching the upper limit of the measurement scale (8, 9), a phenomenon known as the ceiling effect (10, 11). This raises concerns about whether the instruments commonly used are able to detect meaningful differences.

This can depend on the response options used (12). The commonly used Likert style response option contains an equal number of positive and negative response options around a neutral midpoint (e.g., Strongly disagree, neutral, Strongly agree) (13). This allows respondents to express both agreement and disagreement with equal weight and is useful when a similar amount of positive and negative views are expected. It is also possible to have an unbalanced Likert-style response option that contains a greater number of response options on one side, leaning toward positive or negative options (e.g., Not at all, Much, and Very strongly). This allows for finer differentiation in the intensity of responses within the expected direction and is useful when most respondents are anticipated to hold views that are predominantly positive or negative, rather than evenly divided (14). This can help avoid ceiling effects.

One widely used multi-item professional identity scale, The Macleod Clark Professional Identity Scale (MCPIS-9) (3), has demonstrated good reliability and validity in various contexts and has been translated and adapted for use in different cultural and educational settings (15, 16). However, a systematic review found that, although the MCPIS had among the most extensive psychometric evidence of the available professional identity measures (6), it did not meet all COSMIN criteria (17). The MCPIS-9 employs a balanced 5-point Likert response format, comprising two positively and two negatively worded options, and one neutral or undecided option in the centre. Notably, several studies find that students tend to score high on MSPIC-9, even early on in their professional education (18–20). This effect suggests that the MSPIC-9 may not be optimal for capturing differences.

One aspect of multi-item measures, designed to capture latent constructs, such as professional identity, is that they can be time-consuming and burdensome for respondents, contributing to participant fatigue (6). Such practical challenges have prompted growing interest in the use of a single-item question, which are straightforward both to administer and answer (21, 22). Single-item questions have been widely used in various research fields, such as self-reported health status and health-related quality of life (21, 23, 24), emotional exhaustion (25), and job satisfaction (26), where they have demonstrated acceptable reliability and validity and have been recommended for large-scale assessments. Studies in other areas than health and social care education suggest that a single-item question of professional identity can provide practical and psychometrically sound assessments (27–29) Validity and reliability studies of single-item questions of social identification further support their use in research contexts where shortness is essential (30, 31). However, their suitability depends on the nature and breadth of the construct to be measured, the intended use of the measure, and the quality of the individual item (32), and they provide limited information about specific dimensions. Some of the single-item questions use unbalanced 1 to 5 scales as response option (24, 25).

Thus, a single-item question may not capture the range of aspects relevant to a construct to the same extent as multi-item measures. Therefore, it is important to compare single-item and multi-item measures of the same construct to determine whether they yield comparable results.

The aim of this study was therefore to investigate the performance of a single-item question about professional identity with five positively unbalanced response options and a widely used validated one factor 9 item questionnaire with five balanced answers categories (MCPIS-9) on student demographics, educational background, work experience, family educational and occupational history, study choice and academic year. This was explored answering the following research questions:

RQ1. What was the mean and standard deviation of the scores on the MCPIS-9 and the single-item question? This will indicate which measure is more likely to detect differences, reflected by a mean closer to the midpoint and a larger standard deviation.
RQ2. What was the correlation between the MCPIS-9 and the single-item question, and what were the mean scores for the MCPIS-9 on each of the single-item response options? This will indicate whether they measure the same construct and clarify the relationship between the scores.
RQ3. What was the scores on the MCPIS-9 and the single-item question in the different academic years? This will indicate which of the two measures is most responsive to detect difference between students in different academic years.
RQ4. What were the associations between the MCPIS-9 and the single-item question respectively, and student demographics, educational background, work experience, family educational and occupational history, study choice and academic year? This will indicate whether the two measures identify the same variables as being associated with professional identity.
RQ5: What were the associations between the difference in the scores (MCPIS-9 minus the single-item), and student demographics, educational background, work experience, family educational and occupational history, study choice and academic year? This will allow for a statistical test of whether the two measures identify the same variables as being associated with professional identity.

## Methods

This was an explorative cross-sectional study that is part of a larger project comparing interprofessional and professional identity development, utilising the same sample as a previous publication (19).

The study was approved by the Norwegian Agency for Shared Services in Education and Research (SIKT) (reference 701327). Students were provided with written information about the study's purpose, data storage, and protection measures, and consented to participate by submitting the questionnaire. Data were collected in the spring of 2022.

### Setting

The study was conducted at the Norwegian University of Science and Technology (NTNU), which has 13 health and social care study programmes. Approximately 4,800 students are enrolled in these programmes.

### Participants and recruitment

The study aimed to include students from all study years across all health and social care programmes. Study programmes without graduates in 2021 or those not offering a full programme leading to authorization were excluded.

To recruit participants, all eligible students received an email invitation with a link to the digital questionnaire, and most were given information and time to complete the questionnaire during or immediately after a lecture. The questionnaire was available for three months to accommodate the varying times students received information, mainly due to the timing of their practice placements.

### Data collection and variables

A detailed description of the cross-sectional questionnaire study procedure is given elsewhere (19). The questionnaire was made available on the secure survey tool www.nettskjema.no, administered by The University of Oslo, Norway. Two measures of professional identity were used:

‐ *The Norwegian version of the MCPIS-9* (19). The MCPIS-9 is a 9-item validated measure of professional identity. It was selected because it is a brief multi-item instrument that has been used across several health and social care student populations and was available in Norwegian. The response options were a 5-point balanced Likert scale ranging from 1 to 5 (strongly disagree, disagree, neither agree nor disagree, agree, strongly agree) (3). A mean score across all items was calculated for respondents who answered >50% of the items, with higher scores indicating a stronger professional identity.
‐ A *single-item question of professional identity*. The question was self-developed for this study, inspired by previously published single-item measures referenced in the introduction. The aim was to capture students' overall perception of their professional identity using a brief and easily administered measure. The wording and response options were developed by the research group based on theoretical descriptions of professional identity and discussions of potential ceiling effects in existing instruments. It was piloted to assess clarity and comprehensibility. The wording was: “All things considered, to what extent do you identify with the profession you are training for?” (in Norwegian «Alt sett under ett, i hvor stor grad identifiserer du deg med profesjonen du utdanner deg til?»). The response options were a positively unbalanced Likert scale ranging from 1 to 5 (not at all, some¸ much, very much, completely).

To answer RQ5, the difference in score was calculated by subtracting the single item score from the MCPIS-9 mean score, for each student. Thus, positive values indicate that the MCPIS-9 mean score was higher than the single-item question.

The other variables were made from following questions from the questionnaire; age, gender, educational programme, academic year, whether the student was admitted to the study programme they ranked as their first choice (study choice) (yes/no), whether they had worked in health and social care before or during their studies (yes/no), whether their guardians had worked in health and social care (yes/no), and whether any guardian had attained higher education degree (yes/no).

The students' academic year was measured by recoding the variable academic year into three categories: first year, midway (defined as 2nd year for three-year programmes and 2nd—5th year for six-year programmes), and final year.

The length of study programmes was categorized as either three-year bachelor's programmes (audiology, bioengineering, child welfare, nursing, occupational therapy, physiotherapy, radiography, social education, and social work) or six-year professional programmes (medicine and psychology).

### Analyses

The characteristics of the entire sample and the professional identity measures are presented using proportions, frequencies, means, and standard deviations.

Mean and standard deviation (SD) were used to indicate whether there were differences in the variation of the professional identity measures (RQ1), and mean was used to describe the mean scores for the MCPIS-9 on each of the single-item response options (RQ2).

A Spearman rank-order correlation analysis was conducted to examine the relationship between the mean scores of the MCPIS-9 and the single-item question (RQ2).

One-way ANOVA analyses were performed to examine differences in scores between study years (academic year) for all students regardless of study length, as well as for the three- and six-year professional programmes, for both the MCPIS-9 and the single-item question (RQ3).

Three multivariable regression analyses were performed with the dependent variable being either the MCPIS-9 or the single-item question (RQ4), or the difference between the scores (RQ5). The independent variables were student demographics, educational background, work experience, family educational and occupational history, study choice and academic year. The results of the multivariable analyses are presented with adjusted coefficients (adj. coeff.) and 95% confidence intervals (95%CI).

All analyses were conducted using IBM SPSS Statistics version 28.0.1.0.

## Results

Approximately one-fifth of the eligible students (864 individuals) completed the questionnaire. The mean age of respondents was 23.9 years (SD 4.1, median 23), and 77% were female (Table 1). They were from 11 different study programmes, including audiology, bioengineering, child welfare, medicine, nursing, occupational therapy, physiotherapy, psychology, radiography, social education, and social work. About half of the respondents were in either their first or last year of study. Notably, students from medicine, physiotherapy and nursing programmes made up 65% of all the respondents. The only data available for all invited students, was that 81% were women and 65% were between 20 and 24 years old.

**Table 1:** Characteristics of responders. N (%) of the responders within each category. *N* = 864.

Characteristics	N (%)
Academic year	
‐ 1st year student	166 (19.2%)
‐­ Midway student (2nd or 2nd-5th year)	416 (48.1%)
‐­ Final-year student (3rd or 6th year)	278 (32.2%)
Gender	
‐­ Women	668 (77.3%)
‐­ Men	190 (22.7%)
‐­ Non-binary/no answer	4 (0.2%)
Age group	
‐­ < 21	219 (25.3%)
‐­ 22–23	271 (31.4%)
‐­ 24–25	184 (21.3%)
‐­ > 25	186 (21.5%)
Parents/guardians	
‐­ Have higher education degree	706 (81.7%)
‐­ Work within health and social care	366 (42.4%)
The student	
‐­ Worked in health and social care before or during study	453 (52.4%)
‐­ The study programme was the student's first choice	796 (92.1%)
Study programme	
‐­ Audiology	1 (0.1%)
‐­ Bioengineering	46 (5.3%)
‐­ Child welfare	29 (3.4%)
‐­ Medicine	315 (36.5%)
‐­ Nursing	101 (11.7%)
‐­ Occupational therapy	7 (0.8%)
‐­ Physiotherapy	150 (17.4%)
‐­ Psychology	43 (5.0%)
‐­ Radiography	83 (9.6%)
­ Social education	62 (7.2%)
‐­ Social work	26 (3.0%)

### RQ1: distribution of the scores

The overall mean score for the MCPIS-9 with the balanced 5-point scale was 4.15 (SD 0.54) (Table 2).

**Table 2:** Percentage (frequency) of answers to the MCPIS-9 items, and mean scores (standard deviation) *N* = 864.

Statements	Response options					Mean score (SD)
	**Strongly disagree = 1**	**Disagree = 2**	**Neither = 3**	**Agree = 4**	**Strongly agree = 5**	
I feel like I am a member of this profession	1% (8)	3% (25)	17% (145)	54% (469)	25% (214)	**4.0 (0.79)**
I feel I have strong ties with members of this profession	2% (21)	11% (92)	29% (248)	42% (366)	16% (135)	**3.6 (0.96)**
I am often ashamed to admit that I am studying for this profession^a^	1% (4)	3% (29)	8% (72)	26% (223)	62% (535)	**4.5 (0.82)**
I find myself making excuses for belonging to this profession^a^	2% (14)	6% (49)	9% (74)	22% (192)	62% (534)	**4.4 (0.97)**
I try to hide that I am studying to be part of this profession^a^	1% (4)	3% (24)	6% (55)	22% (190)	68% (590)	**4.6 (0.77)**
I am pleased to belong to this profession	1% (5)	1% (8)	8% (69)	42% (360)	49% (419)	**4.4 (0.72)**
I can identify positively with members of this profession	1% (4)	1% (10)	11% (94)	47% (400)	41% (353)	**4.3 (0.73)**
Being a member of this profession is important to me	1% (11)	4% (36)	25% (213)	46% (393)	24% (210)	**3.9 (0.87)**
I feel I share characteristics with other members of the profession	1% (11)	6% (50)	20% (173)	52% (450)	21% (180)	**3.9 (0.86)**
*Mean score across all statements*						***4.15* (*0.54)***

The bold numbers indicate a *P*- value <0.05.

a These statements are originally phrased negatively, but their scores have been reversed to give scores of all the statements the same direction. E.g. for the statement “I am often ashamed…”, 62% scored Strongly disagree, which in the table is presented as Strongly agree.

The mean score for the single-item question on the positively unbalanced 5-point scale was 3.58 (SD 0.99) (Table 3). The distribution of scores along the 1 to 5 range was more distributed for the unbalanced response options in the single-item question than for the scores on the balanced MCPIS-9 scale. This is reflected in the size of the standard deviation (i.e., variation in answers), which was nearly twice as large for the single-item question (SD = 0.99) than for MCPIS-9 (SD = 0.54).

**Table 3:** Distribution of answers to the single-item question, and the mean score and standard deviation (SD). *N* = 863.

Question	Response option [proportion (N)]					Mean score (SD)
	Not at all = 1	Some = 2	Much = 3	Very much = 4	Completely = 5	
All things considered, to what extent do you identify with the profession you are training for?	1% (9)	16% (140)	24% (210)	41% (351)	18% (153)	3.58 (0.99)

### RQ2: correlation between the measures

There was a significant correlation between MCPIS-9 and the single-item question (Spearman's rho = 0.605, *p* < 0.001).

The MCPIS-9 mean scores ranged from 3.0 among students who reported feeling that they “not at all” belong to their chosen profession on the single-item question, to 4.6 among those who felt they “completely” belong (Figure 1).

**Figure 1:**
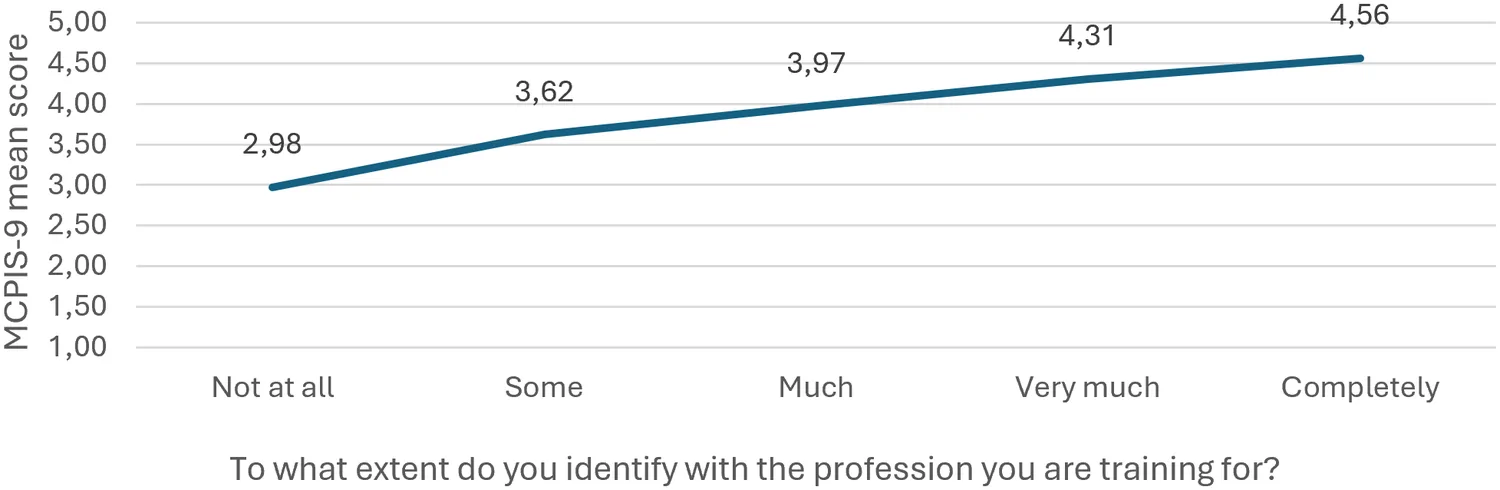
The mean MCPIS-9 score for each single-item question response option.

### RQ3: the MCPIS-9 and the single-item question in different academic years

The mean professional identity scores were higher in the final year compared to the first year by 0.19 points for the MCPIS-9 score (4.23 vs. 4.04 *p* < 0.001) and by 0.68 points for the single-item question (3.84 vs. 3.16) (Table 4).

**Table 4:** Professional identity during education assessed by the MCPIS-9 and a single-item question, as well as the difference between the two scores.

Academic year	MCPIS-9 (*N* = 860) Mean (95% CI)	Single-item (*N* = 859) Mean (95% CI)
1st year	4.04 (3.95 to 4.12)	3.16 (2.99 to 3.32)
Midway (2nd or 2nd-5th year)	4.13 (4.08 to 4.18)	3.57 (3.48 to 3.66)
Final year (3rd or 6th year)	4.23 (4.17 to 4.30)	3.84 (3.73 to 3.95)

When analysed separately for students in three- and six-year professional study programmes (Figure 2), the difference between first and final year students in the single-item question was 0.62 points in the three-year programmes and 0.86 points in the six-years programmes (both *p* < 0.001). For the MCPIS-9, the corresponding difference was 0.16 points for the three-year programmes (*p* = 0.034) and 0.30 points for the six-year programmes (*p* = 0.022).

**Figure 2:**
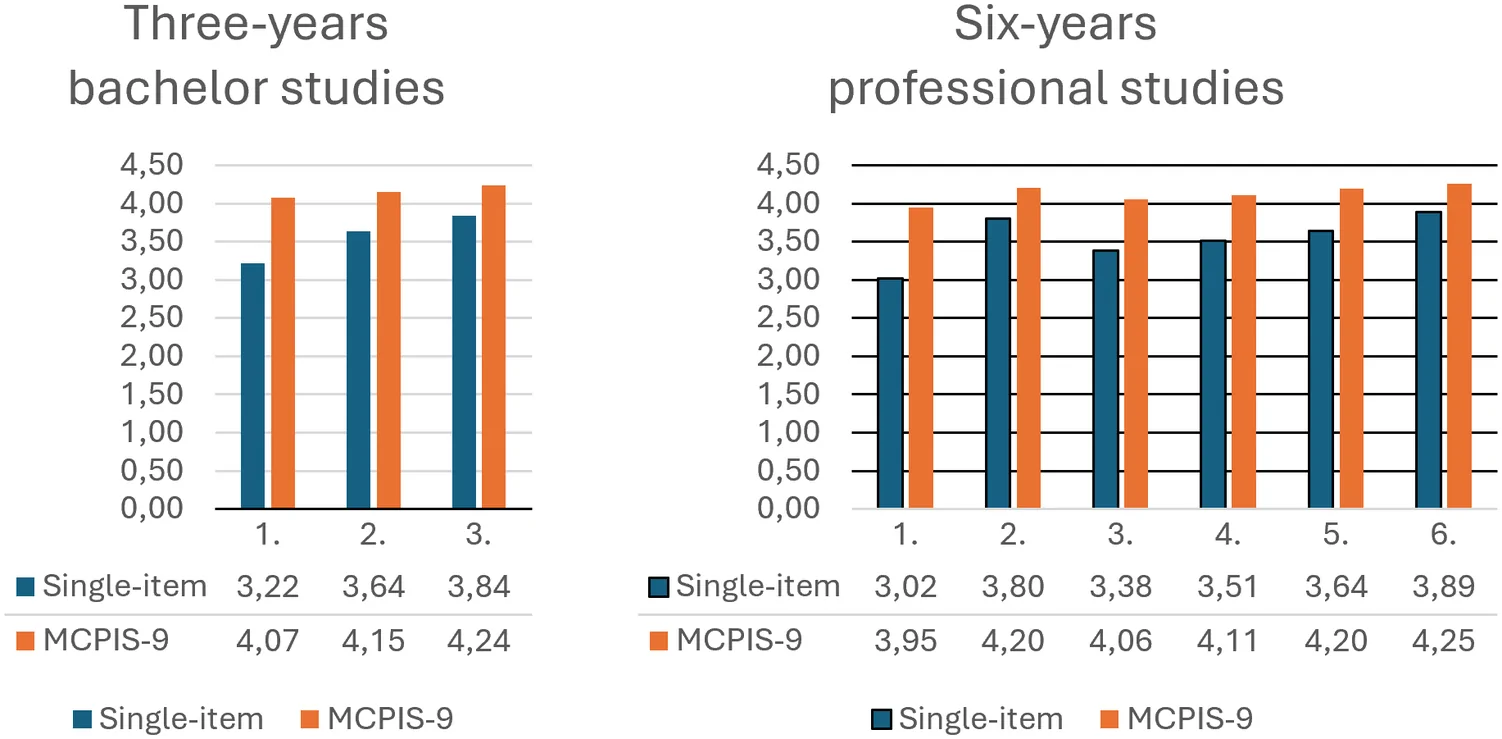
Professional identity score for the single-item question and the MCPIS-9 for each study year for three-years and six-years studies respectively. Mean scores.

### RQ4: associations between the MCPIS-9 and the single-item question score respectively, and student characteristics

Table 5 presents the results from the multivariable regression models for MCPIS-9 and the single-item question, respectively. In summary, the direction of the adjusted coefficient for the variables that had an influence on professional identity, were the same in both models. In both models, being in a higher academic year of the professional programme and having had the professional programme as ones first choice was associated with higher professional identity.

**Table 5:** Mean scores (standard deviation, SD) and adjusted coefficients (Adj.coeff) from multivariable regression analyses for MCPIS-9 and single-item question scores respectively, adjusted for student characteristics. A positive adjusted coefficient is associated with higher professional identity.

Variable	MCPIS-9 scale (*N* = 860)^a^			Single-item question (*N* = 859)^b^		
	Mean score (SD)	Adj. coeff. (95% CI)	*P*- value	Mean score (SD)	Adj. coeff. (95% CI)	*P*- value
Academic year						
‐­1st year	4.04 (0.55)	REF		3.16 (1.08)	REF	
‐­Midway	4.13 (0.53)	0.07 (−0.02 to 0.17)	.138	3.57 (0.94)	**0.36 (0.19 to 0.54)**	**<** **.** **001**
‐­Final year	4.23 (0.52)	**0.18 (0.07 to 0.30)**	**<** **.** **001**	3.84 (0.93)	**0.63 (0.43 to 0.83)**	**<** **.** **001**
Gender						
‐­Men	4.18 (0.52)	REF		3.72 (1.00)	REF	
‐­Women	4.14 (0.54)	-0.03 (−0.11 to 0.06)	.538	3.54 (0.99)	**-0.16 (−0.32 to 0.0)**	**.** **049**
Age group:						
‐­≤ 21 years	4.12 (0.50)	REF		3.40 (0.97)	REF	
‐­22–23 years	4.12 (0.53)	−0.05 (−0.15 to 0.05)	.314	3.55 (0.95)	−0.04 (−0.22 to 0.14)	.688
‐­24–25 years	4.17 (0.57)	−0.01 (−0.11 to 0.11)	.932	3.65 (1.05)	0.01 (−0.19 to 0.21)	.935
‐­≥ 26 years	4.20 (0.55)	−0.01 (−0.12 to 0,11)	.906	3.77 (1.01)	0.05 (−0.16 to 0.25)	.648
Guardians have higher education degree						
‐­No	4.21 (0.51)	REF		3.72 (1.03)	REF	
‐­Yes	4.13 (0.54)	−0.07 (−0.16 to 0.02)	.149	3.54 (0.98)	−0.13 (−0.30 to 0.04)	.129
Guardians work in health and social care						
‐­No	4.14 (0.52)	REF		3.55 (1.02)	REF	
‐­Yes	4.15 (0.55)	0.02 (−0.06 to 0.09)	.674	3.61 (0.96)	−0.06 (−0.07 to 0.19)	.366
Student had worked in health and social care before study						
‐­No	4.11 (0.56)	REF		3.60 (1.01)	REF	
‐­Yes	4.18 (0.51)	**0.08 (0.00 to 0.15)**	**.** **043**	3.57 (0.98)	−0.01 (−0.14 to 0.13)	.911
Student had study programme as 1st choice						
*No*	3.80 (0.69)	REF		3.10 (1.12)	REF	
*Yes*	4.17 (0.51)	**0.36 (0.23 to 0.50)**	**<** **.** **001**	3.61 (0.97)	**0.47 (0.23 to 0.71)**	**<** **.** **001**

The bold numbers indicate a *P*- value <0.05.

a The model accounts for 5% of the variance.

b The model accounts for 7% of the variance.

The adjusted coefficients for these variables were larger for the single-item question. For academic year, the professional identity score for final-year students was 0.63 points higher than for first year students on the single-item question, while it was 0.18 on the MCPIS-9. For students who had their study programme as their first choice, the professional identity score was 0.47 points for the single-item question, and 0.36 for MCPIS-9.

For two other variables, there were statistically significant but small adjusted coefficients that differed in the two models. Being female was statistically significantly associated with lower professional identity only on the single-item question, although the direction of the association was the same for the MCPIS-9 (i.e., lower scores among females). For the MCPIS-9, there was a statistically significant association between higher professional identity and the student having worked in health and social care. No such association was observed for the single-item question.

### RQ5: associations between difference in the MCPIS-9 and the single-item question score, and student characteristics

Similar to the overall difference in mean score between the two measures (0.57 = the mean MCPIS-9 score 4.15 minus the single-item question score 3.58), the single-item question score was consistently lower for all variables in the multivariable analysis with the difference between the two scores as the dependent variable (Table 6).

**Table 6:** Mean scores (standard deviation, SD), mean difference (SD) in the score of MCPIS-9 minus the single-item score, change in mean difference (how the calculation was done) and the adjusted coefficients (Adj.coeff) with 95% confidence interval (95%CI) from multivariable regression analyses with the difference in the score of MCPIS-9 minus the single-item score as dependent variables and student characteristics as the independent variables.

Variable	MCPIS-9 (SD)	Single-item (SD)	Mean difference (SD)	Difference between MCPIS-9 and single-item (*N* = 859)		
				Change in mean difference^a^	Adj. coeff. (95% CI)	*P*-value
Academic year						
1st year	4.04 (0.55)	3.16 (1.08)	0.88 (0.85)	-	REF	
‐­Midway	4.13 (0.53)	3.57 (0.94)	0.56 (0.79)	−0.32 (=0.56 minus 0.88)	**−0.29 (−0.44 to −0.15)**	**<** **.** **001**
‐­Final year	4.23 (0.52)	3.84 (0.93)	0.39 (0.70)	−0.49 (=0.39 minus 0.88)	**−0.45 (−0.61 to −0.29)**	**<** **.** **001**
Gender						
‐­Men	4.18 (0.52)	3.72 (1.00)	0.46 (0.75)	-	REF	
‐­Women	4.14 (0.54)	3.54 (0.99)	0.60 (0.80)	0,14 (=0.60 minus 0.46)	**0.13 (0.00 to 0.26)**	**.** **045**
Age group						
‐­≤ 21 years	4.12 (0.50)	3.40 (0.97)	0.71 (0.81)		REF	
‐­22–23 years	4.12 (0.53)	3.55 (0.95)	0.57 (0.77)	−0,14 (=0.57 minus 0.71)	−0.01 (−0.16 to 0.13)	.872
‐­24–25 years	4.17 (0.57)	3.65 (1.05)	0.53 (0.81)	−0,18 (=0.53 minus 0.71)	−0.01 (−0.17 to 0.15)	.886
‐­≥ 26 years	4.20 (0.55)	3.77 (1.01)	0.44 (0.77)	−0,27 (=0.44 minus 0.71)	−0.05 (−0.22 to 0.11)	.532
Guardians have higher education degree						
‐­No	4.21 (0.51)	3.72 (1.03)	0.49 (0.85)	-	REF	
‐­Yes	4.13 (0.54)	3.54 (0.98)	0.59 (0.78)	0,10 (=0.59 minus 0.49)	0.07 (−0.07 to 0.20)	.352
Guardians work in health and social care						
‐­No	4.14 (0.52)	3.55 (1.02)	0.59 (0.73)	-	REF	
‐­Yes	4.15 (0.55)	3.61 (0.96)	0.53 (0.79)	−0,06 (=0.53 minus 0.59)	−0.05 (−0.15 to 0.06)	.410
Student had worked in health and social care before study						
‐­No	4.11 (0.56)	3.60 (1.01)	0.52 (0.79)	-	REF	
‐Yes	4.18 (0.51)	3.57 (0.98)	0.61 (0.80)	0,11 (=0.61 minus 0.52)	0.08 (0.14 to −0.03)	.135
Student had study programme as 1st choice						
‐No	3.80 (0.69)	3.10 (1.12)	0.70 (0.78)	-	REF	
‐­Yes	4.17 (0.51)	3.61 (0.97)	0.56 (0.79)	−0,14 (=0.56 minus 0.70)	−0.11 (−0.31 to 0.09)	.279

The bold numbers indicate a *P*- value <0.05.

The model accounts for 5% of the variance.

a The change in mean difference is calculated to make it easier to see the difference between the observed values and the adjusted coefficients (i.e., the influence of the adjustment).

The adjusted coefficients in Table 6 shows whether the two measures differed in their ability to detect differences. If there were no significant difference, the two measures had the same ability to detect differences. Significant differences, regardless of whether adjusted coefficient were positive or negative, shows that one measures had a greater ability to detect differences.

Given the larger variation in the scores of the single-item question, the finding of significant differences in the adjusted coefficients confirms that the single-item question has the best ability to detect differences.

The largest difference between the two measures was observed for academic year, where the adjusted size of the difference was 0.45 points (95%CI (0.29 to 0.61, *p* < 0.001) (Table 6).

## Discussion

The main finding was that the single-item question and the MCPIS-9 showed similar patterns of association with key student characteristics; professional identity was higher among final-year students and among those who had the profession as their first study choice. Of the two measures, the single-item question showed a better ability to differentiate.

### Do they measure the same construct?

The key question is whether the two measures assess the same construct. To address this, concurrent criterion validity and convergent validity, which assess whether a new measure aligns with an established criterion, are used (32). Concurrent criterion validity and convergent validity are typically evaluated by the strength and coherence of the association, together with evidence that they relate similarly to relevant external variables. In this study, the single-item question and MCPIS-9 were administered together and showed a clear, statistically significant, moderate correlation. Furthermore, they showed the same directional relationships in the multivariable regression analysis. Taken together, these findings give preliminary evidence of the concurrent and convergent validity of the single-item question relative to the MCPIS-9.

As the single-item question is substantially easier to administer and understand for respondents (21, 22, 32), it is reasonable to argue that it is the better choice when the goal is to obtain an overall estimate of professional identity. A single-item question reduce respondent burden and survey length, lowering fatigue and administration costs while still providing acceptable validity evidence in large-scale settings (21, 23–26). Postmes et al. (31) have developed a single-item social identification measure (SISI) involving rating one's agreement with the statement “I identify with my group (or category)”. These authors conclude that social identification can be measured with a single item (31). Together with the concurrent criterion and convergent validity evidence above, the practical advantages make the single-item question a defensible first choice for routine monitoring of overall professional identity. Whether longer multi-item scales are needed for more detailed assessments remains an open question.

### Using balanced or unbalanced response options?

A major difference between the two measures used in this study was the response options. The MCPIS-9 has a balanced 5-point Likert format, whereas the single-item question had a positively unbalanced scale with finer gradations at the endorsement end. As outlined in the introduction, positively unbalanced scales are suitable when most respondents report high agreement, which creates a real risk of ceiling or near ceiling clustering on balanced scales.

The question is whether this is the case for professional identity. Studies have found that students often report high professional identity, also already early in training. For example, paramedic students reported a high mean MCPIS-9 (38.8 of a maximum score of 45, similar to 4.3 on a 1 to 5 scale) with limited dispersion and only year of study predicting variation (20). Another study among pharmacy students also using MCPIS-9 found the same level of professional identity across all four study years, concluding that the overall level of PI among student pharmacists was high (9). Furthermore, these observations match the current study, where the MCPIS-9 also showed similar levels. However, there are studies that have found a lower mean score on the MCPIS-9, like in Chinese therapy students (15). Still, taken together, the literature seems to strongly support the notion that health and social care students report high professional identity.

The next question is whether a positively unbalanced scale is a better alternative. The Standards for Educational and Psychological Testing emphasize selecting formats that fit the intended use and the population's response distribution as part of a cumulative validity argument, rather than treating one response format as universally superior (33). In health research, single-item questions with positively unbalanced scales measuring self-rated health have long been used (34). The evidence for its value was confirmed already in a review from 1997 (35) and later a meta-analysis from 2006 finding that such questions have a strong association with mortality also after adjustment for relevant covariates (36).

The finding in this study that the positively unbalanced response options used in the single-item question produced lower means and higher variance than the MCPIS-9, indicates that this might be the case also for professional identity. However, more independent studies are needed to confirm this.

Furthermore, the greater score variability and stronger differentiation observed for the single-item question cannot be attributed solely to the positively unbalanced response options. The two instruments also differed in the number of items, item wording, and the aspects of professional identity being assessed.

### Strengths and limitations

A key strength of this study is the inclusion of a relatively large and heterogeneous sample. Comparing the MCPIS-9 with a theoretically grounded single-item alternative within the same dataset provides a strong empirical basis for the analyses.

The cross-sectional design precludes conclusions about development over time. Differences observed between academic years can thus be due to cohort effects (differences between the students in the different academic years). Furthermore, the response rate was approximately one-fifth of the eligible population, which raises the possibility of non-response bias, although key demographic characteristics were broadly comparable to the underlying student population. In addition, participation varied considerably between programmes, with most respondents coming from medicine, physiotherapy, and nursing, while some programmes were represented by relatively few students. The findings should therefore be interpreted with caution for individual programmes. Thirdly, the observed difference between the single-item measure and MCPIS-9 can be due to other things than the response format, like the content and wording of the item or other measurement characteristics. Finally, the findings may not be directly generalisable beyond similar educational settings and contexts, and replication in other universities and countries is needed to assess the transferability of the findings.

## Conclusion

The single-item measure showed comparable validity evidence to the MCPIS-9 and produced greater score dispersion. Given its simplicity and low respondent burden, it may be a useful alternative as and overall measure of professional identity.

## Data Availability

The data analyzed in this study is subject to the following licenses/restrictions: Available upon reasonable request to the first author. Requests to access these datasets should be directed to aslak.steinsbekk@ntnu.no.
